# Standardization of polymerase chain reaction for detection of fluconazole resistance targeting Y132F mutation in *ERG11* gene in *Candida parapsilosis*

**DOI:** 10.22034/cmm.2024.345209.1517

**Published:** 2024-05-07

**Authors:** Kanagasabapathi Karthika, Thayanidhi Premamalini, Thanneru Vijayakishore, Anupama Jyoti Kindo

**Affiliations:** Department of Microbiology, Sri Ramachandra Medical College & Research Institute, SRIHER, Porur, Chennai 600116, India

**Keywords:** *Candida parapsilosis*, *ERG11* gene, Fluconazole resistance, PCR, Y132F mutation

## Abstract

**Background and Purpose:**

*Candida parapsilosis* is the third most commonly isolated species from candidemia patients admitted to Indian intensive care units. Outbreak of infection and emergence of fluconazole resistance associated with this particular species has been increasingly documented since 2018. Worldwide data has documented that Y132F substitution in the *ERG11* gene is the predominant fluconazole resistance mechanism among *C parapsilosis*. Hence, this study aimed to detect fluconazole resistance by targeting Y132F mutation in the *ERG11* gene in *C. parapsilosis*, by conventional polymerase chain reaction (PCR) assay with in-house designed primers.

**Materials and Methods:**

A total of 75 *Candida* isolates were collected from candidemia patients (Jan-Dec 2023). All the *Candida* isolates were subjected to phenotypic and genotypic characterization. PCR-restriction fragment length polymorphism was performed for identification and confirmation of *C. parapsilosis* isolates. The antifungal susceptibility testing by broth microdilution method was performed according to the Clinical and Laboratory Standards Institute guidelines (M27-A3) for all *C. parapsilosis* against fluconazole, itraconazole, voriconazole, and posaconazole to determine their minimum inhibitory concentration (MIC) values. *Candida parapsilosis*-specific PCR assay was developed with in-house designed primers to detect Y132F mutation in the *ERG11* gene.

**Results:**

In this study, among 75 candidemia patients (Jan-Dec 2023), about 24% of the candidemia was caused by *C. parapsilosis*. Fluconazole resistance among *C. parapsilosis* was found to be 16.7% with a MIC range of 32–64 µg/ml. The PCR assay successfully identified all three fluconazole-resistant *C. parapsilosis* with Y132F mutation, thereby confirming the PCR results. Furthermore, validation of the presence and absence of Y132F mutation in resistant and susceptible isolates by DNA sequencing showed that the results were in concordance with our PCR assay.

**Conclusion:**

The developed PCR assay successfully detected the Y132F mutation within 3 h. This assay can be useful for early detection of fluconazole-resistant *C. parapsilosis* isolates in candidemia patients, which helps the provision of early antifungal treatment for better patient management.

## Introduction

Over the past decade, the incidence of *Candida parapsilosis* has dramatically increased. In Asia, Latin America, and Southern Europe, *C. parapsilosis* is a major cause of invasive candidiasis and even outranks *Candida albicans* in some European, Asian, and South American hospitals [ 1 , 2 ]. *Candida parapsilosis* is encountered in multiple environments due to its unique ability to grow on inanimate objects and surfaces. It causes nosocomial spread by healthcare workers via hand carriage and also forms biofilms on catheters and other implanted devices [ 3 , 4 , 5 ]. *Candida parapsilosis* is of special concern in critically ill neonates, where it is often associated with mortality [ 6 ]. The number of reports on *C. parapsilosis* outbreaks has increased in the last decade and also these outbreaks are no longer limited to a specific patient population. In many developing countries, fluconazole is the most widely used antifungal agent for the treatment of *Candida* infections. Although *C. parapsilosis* was initially considered fully susceptible to fluconazole, the emergence of fluconazole-resistant *C. parapsilosis* has been reported in many countries in the last few years. Fluconazole prevents fungal cell growth by inhibition of lanosterol 14-alpha demethylase, a protein encoded by the *ERG11* gene, which leads to a blockage of ergosterol synthesis, an essential component of fungal cell membranes. Fluconazole-resistant strains might replace susceptible *C. parapsilosis* isolates, leading to severe outbreaks that could persist despite the application of strict infection control strategies and may cause high mortality in patients with invasive disease. Several reports suggest that fluconazole resistance in *C. parapsilosis* may emerge following drug pressure in the form of fluconazole treatment [ 7 , 8 ]. The fluconazole resistance mechanisms include increased expression of target of azole, efflux of drug from the cell, point mutations in the *ERG11* gene, overexpression of *ERG11*, and development of bypass pathways. Fluconazole resistance is mainly influenced by amino acid substitutions, although the data available is very limited. The main mechanism related to fluconazole resistance in *C. parapsilosis* is the presence of *ERG11* mutations, dominated by Y132F amino acid substitution which has been described earlier in *C. albicans*, *C. tropicalis*, and *C. auris* [ 9 , 10 , 11 ]. *Candida parapsilosis* isolates harboring Y132F amino acid substitution have been recently reported in several countries. Isolates harboring this substitution mimic *C. auris*, they may cause hospital outbreaks, become endemic, and emerge simultaneously in distant areas around the world [ 1 ]. This study is the first of its kind in India to rapidly detect fluconazole resistance by targeting Y132F mutation among *C. parapsilosis* isolates by using in-house primers.

## Materials and Methods

### Isolation and identification of Candida parapsilosis isolates

In total, 75 positive blood cultures that grew yeast were collected from candidemia patients over a period of 1 year (Jan-Dec 2023) from a tertiary care center. The isolates were initially identified by conventional phenotypic techniques based on gram staining, culture characteristics, germ tube formation, sugar fermentation, sugar assimilation, color on Tetrazolium reduction medium (TTZ), and color on CHROM agar with appropriate control strains. All the isolates that were identified phenotypically were further confirmed by genotypic identification techniques, such as polymerase chain reaction-restriction fragment length polymorphism (PCR-RFLP) [ 12 ].

### DNA extraction

DNA was extracted from all the *Candida* isolates by the Phenol-Chloroform method with minor modifications [ 12 , 13 ]. The extracted DNA was stored at -20°C for further molecular assays. 

### Polymerase Chain Reaction

The PCR amplification of the internal transcribed spacer region (ITS 1 and ITS2) was carried out using universal fungal primers ITS 1 (5’ °– TCC GTA GGT GAA CCT GCG G – 3’) and ITS 4 (5’ – TCC TCC GCT TAT TGA TAT GC – 3’). The PCR master mix was prepared containing 25 μl of PCR mix (Takara, Japan), 1 μl of forward and reverse primer, 5 μl of template DNA, and the volume was increased with sterile nuclease‑free water to reach 50 μl. The reaction mixtures were amplified in a thermal cycler (Veriti 96 well, Applied Biosystems, USA), with the following reaction conditions: at 95 °C for 5 min, followed by 35 cycles at 95 °C for 30 sec, 56 °C for 30 sec, and 72 °C for 30 sec, with a final extension period at 72 °C for 10 min. After amplification, 10 μl of the PCR product was subjected to gel electrophoresis in 1.5% agarose gel, stained with ethidium bromide, and visualized under ultraviolet illumination in a Bio-Rad Gel Documentation system (USA).

### Restriction Fragment Length Polymorphism

The PCR products were subjected to restriction analysis using *Msp* I restriction enzyme (New England Biolabs) which cleaved the DNA at a specific site, producing fragments of varying length [ 13 , 14 ]. The species were identified based on the band size. The prepared reaction mixture contained 10 μl of amplified PCR product, 2 μl of 10X enzyme buffer, and 5 units of *Msp* I restriction enzyme, and the volume was increased with sterile nuclease‑free water to reach 20 μl. The reaction mixtures were incubated at 37 ºC for 1 hour. After incubation, 10 μl of the amplified products were subjected to gel electrophoresis in 2% agarose gel, stained with ethidium bromide, and visualized under ultraviolet illumination in a Bio-Rad Gel Documentation system (USA).

### Antifungal Susceptibility Testing (AFST)

Antifungal susceptibility testing was performed for all *C. parapsilosis* isolates by broth microdilution method, according to the Clinical and Laboratory Standards Institute (CLSI) guidelines (M27-A3) [ 14 ]. The minimum inhibitory concentrations of fluconazole (0.25-64 µg/ml), itraconazole (0.06-16 µg/ml), voriconazole (0.06-16 µg/ml), and posaconazole (0.06-16 µg/ml) were determined after 24 h of incubation at 37°C. Isolates with MIC≥8 was considered as fluconazole-resistant based on CLSI M27M44S-ED3:2022 [ 15 ]. *Candida parapsilosis* ATCC 22019 was used as the reference strain. 

### Standardization of polymerase chain reaction for detection of Y132F mutation in ERG11 gene

Extracted DNA from all *C. parapsilosis* isolates, irrespective of their antifungal susceptibility pattern, was used for the detection of Y132F mutation in the *ERG11* gene. Standardization of PCR was performed using in-house primers targeting Y132F mutation in the *ERG11* gene in *C. parapsilosis*. Using these primers, PCR was standardized for the detection of fluconazole resistance. *Candida parapsilosis* ATCC 22019 was used as a reference strain since it carried the wild-type *ERG11* codon 132 (Y132).

### Primer designing

The nucleotide sequences of the *ERG11* gene of *C. parapsilosis* were obtained from the NCBI database and were aligned using MEGA software (version 11). *Candida parapsilosis* ATCC *ERG11* gene (GQ302972.1) and *C. parapsilosis* LEMI8646 (KR082784.1) [ 16 ] were used as reference strains for primer designing. The primers were designed manually by targeting the mutation at 395 nucleotide position in the *ERG11* gene where Adenine was replaced with Thymine which resulted in Y132F amino acid substitution in the protein. The designed primers were verified by the NCBI Primer-BLAST tool and further analyzed by in-silico PCR (http://insilico.ehu.es/user_seqs/) software. The primers were synthesized by Sigma-Aldrich Chemical Pvt. Ltd., Bangalore.

### Polymerase chain reaction amplification for detection of Y132F mutation in ERG11 gene

The PCR amplification was carried out using the designed primer, CP Y132FF 5’- TTCGATTGTCCGAATGCAAGAC-3’as forward primer, and CP Y132FR 5’- GTTGCCACCTTTACCAGATA-3’ as the reverse primer. The reaction mix was prepared which contained 15 μl of PCR mix (Takara, Japan), 0.5 μl of forward and reverse primer, and 5 μl of DNA template, and the volume was increased to 30 μl with sterile nuclease‑free water. The following were the PCR reaction conditions for the detection of Y132F mutation in the *ERG11* gene: 95 °C for 10 min, followed by 40 cycles consisting of 95 °C for 30 sec, 56 °C for 30 sec, and 72 °C for 30 sec, with a final extension period at 72 °C for 7 min. After amplification, 10 μl of the PCR products were subjected to gel electrophoresis in 1.5% agarose gel, stained with ethidium bromide, and visualized under ultraviolet illumination in a Bio-Rad Gel Documentation system (USA).

### Comparison of AFST and Y132F mutation detecting polymerase chain reaction

The antifungal susceptibility pattern of all *C. parapsilosis* isolates was compared with the results of PCR for rapid detection of fluconazole-resistant using in-house primers specific for Y132F mutation in the *ERG11* gene. Furthermore, it was validated for the effectiveness of the PCR by performing gene sequencing of the *ERG11* gene for Y132F mutation.

### ERG11 gene amplification

The DNA extracted from both fluconazole-susceptible and resistant isolates of *C. parapsilosis* was used as the template. The PCR amplification of *ERG11* gene was carried out using CP_ERG11_F1 5’ -CGAGATAATCATCA ACGAACATTC- 3’ and CP_ERG11 _R2 5’ - AAAGACCGCATTGACTACCGAT-3’ primers [ 16 ]. The reaction mix was prepared which contained 10 μl of PCR mix (Takara, Japan), 0.5 μl of forward and reverse primer, and 3 μl of template DNA, and the volume was increased with sterile nuclease‑free water to reach 20 μl. The reaction mixtures were amplified in a thermal cycler (Veriti 96 well, Applied Biosystems, USA) with the following reaction conditions: 95 °C for 10 min, followed by 35 cycles at 95 °C for 45 sec, 56 °C for 30 sec, and 72 °C for 90 sec, with a final extension period at 72 °C for 10 min. After amplification, 10 μl of the PCR products were subjected to gel electrophoresis in 1.5% agarose gel, stained with ethidium bromide, and visualized under ultraviolet illumination in a Bio-Rad Gel Documentation system (USA).

### Sequencing of ERG11 gene and Y132F mutation analysis

The *ERG11* PCR products of fluconazole susceptible and resistant isolates were sequenced (Eurofins Genomics India Pvt. Ltd., Bengaluru, Karnataka). The DNA sequences obtained were verified by the Basic Local Alignment Search Tool (BLAST) analysis and deposited at the National Centre for Biotechnology Information (NCBI, USA – http://www.ncbi.nlm.nih.gov) GenBank online database and provided with sequence accession numbers. The nucleotide sequences were then translated to amino acid sequences using the BioEdit tool with standard genetic code as a reference. The sequences were aligned using the Clustal Omega program at http://www.ebi.ac.uk/Tools/msa/clustalo/ and analyzed for Y132F amino acid substitution in the protein.

### Ethical Considerations

This study was approved by the Ethics Committee of Sri Ramachandra Institute of Higher Education and Research, India (IEC-NI/20/FEB/74/10).

## Results

Out of all (n=75) *Candida* isolates, *C. albicans* accounted for about 33.3% (n=25), followed by *C. tropicalis* (26.7%, n=20), *C. parapsilosis* (24%, n=18), *C. auris* (8%, n=6), *C. glabrata* (5.3%, n=4), *C. krusei* (1.3%, n=1), and *C. orthopsilosis* (1.3%, n=1).

### Interpretation of antifungal susceptibility testing

In total, 18 *C. parapsilosis* isolates were tested by broth microdilution method, three of which (16.7%) were resistant to fluconazole with MIC values of 32–64 µg/ml. All three *C. parapsilosis* isolates were resistant to fluconazole alone and susceptible to all other antifungal agents (itraconazole, voriconazole, and posaconazole). The remaining 15 *C. parapsilosis* isolates were susceptible to all antifungal agents.

### Detection of Y132F mutation in ERG11 gene

**Figure 1:**
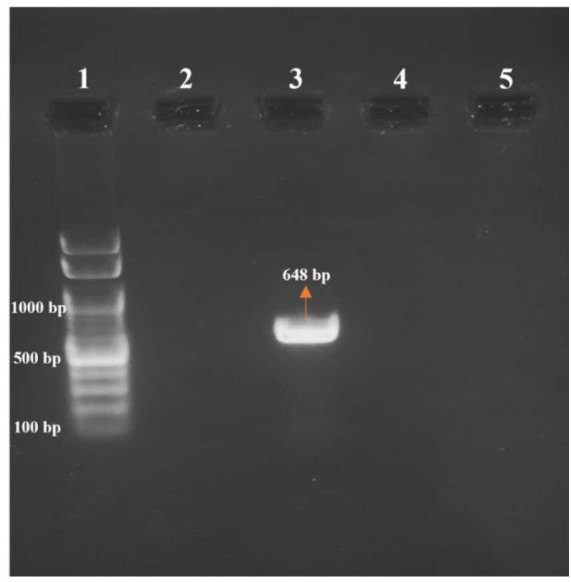
Detection of Y132F mutation in *ERG11* gene of *Candida parapsilosis*. Lane 1: DNA marker (100bp) Lane 2: *Candida parapsilosis* ATCC 22019 Lane 3: *C. parapsilosis* with Y132F mutation (fluconazole resistant) Lane 4: *C. parapsilosis* without Y132F mutation (fluconazole sensitive)

The PCR amplification was performed for all *C. parapsilosis* isolates using self-designed Y132F mutation-detecting primers and the amplified product was subjected to gel electrophoresis. *Candida parapsilosis* resistant isolates with Y132F mutation yielded a band size of 648bp, whereas no band was observed for all susceptible *C. parapsilosis* isolates without Y132F mutation (Figure 1). The PCR assay developed for the detection of the Y132F mutation in the *ERG11* gene identified all 3 *C. parapsilosis* isolates known to carry this mutation. The assay successfully identified the mutation in fluconazole-resistant isolates. 

### Comparison of AFST and Y132F mutation detecting polymerase chain reaction

*Candida parapsilosis* isolates with Y132F mutation which were resistant to fluconazole by broth microdilution method produced band, whereas *C. parapsilosis* isolates without Y132F mutation which were susceptible to fluconazole did not produce any band when specifically designed mutation detecting primers were used.

### ERG11 gene amplification

**Figure 2:**
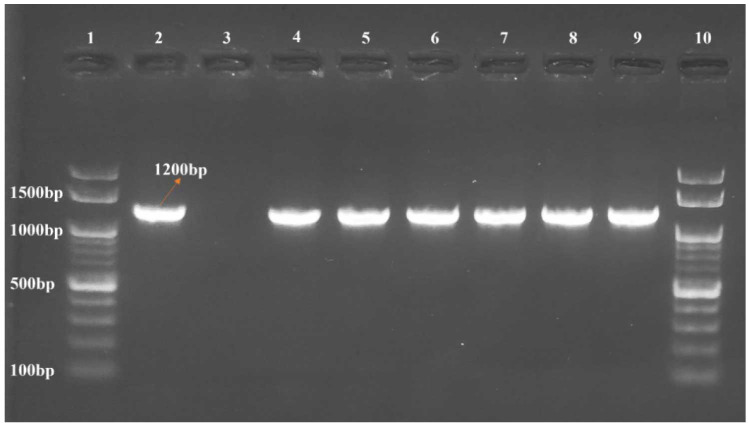
Amplification of *ERG11* gene of *Candida parapsilosis* Lane 1: DNA marker (100bp), Lane 2: Positive control (*C. parapsilosis* ATCC 22019), Lane 3: Negative control (Nuclease free water), Lane 4: *C. parapsilosis* clinical isolates (fluconazole-resistant), Lane 5: *C. parapsilosis* clinical isolates (fluconazole-resistant), Lane 6: *C. parapsilosis* clinical isolates (fluconazole-resistant), Lane 7: *C. parapsilosis* clinical isolates (fluconazole-sensitive), Lane 8: *C. parapsilosis* clinical isolates (fluconazole-sensitive), Lane 9: *C. parapsilosis* clinical isolates (fluconazole-sensitive), Lane 10: DNA marker (100bp)

The *ERG11* gene from all fluconazole susceptible and resistant isolates were amplified. The *ERG11* gene was approximately 1200bp long as shown in Figure 2.

### Y132F mutation analysis in ERG11 gene

**Figure 3:**
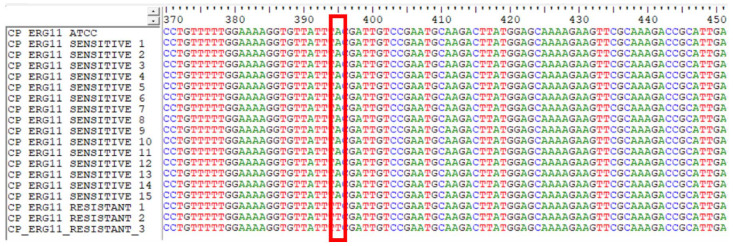
Multiple sequence alignment of *ERG11* gene of susceptible and resistant *Candida parapsilosis* isolates

**Figure 4:**
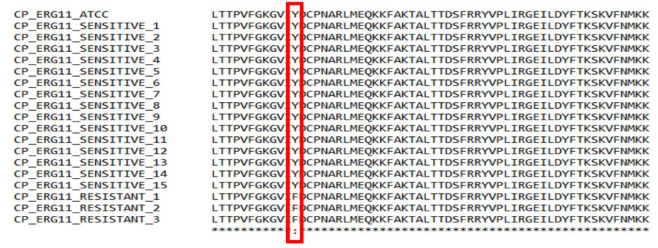
Multiple sequence alignment of *ERG11* gene of susceptible and resistant *Candida parapsilosis* isolates

The presence of Y132F mutation in fluconazole-resistant *C. parapsilosis* isolates were confirmed by sequencing the amplified PCR product of the *ERG11* gene. The *ERG11* gene from all fluconazole susceptible and resistant isolates were amplified and sequenced. *ERG11* sequences from each isolate were compared to *C. parapsilosis* ATCC 22019 *ERG11* [GenBank accession number GQ302972]. The nucleotide sequences were then translated to amino acid sequences and analyzed for Y132F amino acid substitution in the protein (*i.e.*, the tyrosine in the 132nd position was replaced by phenylalanine). The Y132F amino acid substitution was found in all three resistant isolates, whereas it was not found in susceptible isolates (Figures 3 and 4).

### Nucleotide sequence accession numbers

The sequences obtained in this study have been provided with the following GenBank accession numbers - PP054560, PP069229, PP069236-PP069239, PP342300- PP342311.

DiscussionAntifungal susceptibility testing by broth microdilution method remains the gold standard for detection of fluconazole resistance, but it is not widely used in routine diagnostic laboratories since it is time-consuming, expensive, and laborious. Given the rising fluconazole resistance in *C. parapsilosis*, the development of alternative molecular approaches that can quickly determine antifungal resistance, like PCR, is imperative. This will be a rapid, reliable, accurate, and cost-effective method for optimum selection of antifungal therapy to aid better patient management. Therefore, in the present study, this PCR assay using in-house primers to detect Y132F mutation will aid in the recognition of fluconazole resistance in *C. parapsilosis*.

In the present study, *C. parapsilosis* isolates accounted for about 24% of the candidemia cases, being the third most predominant *Candida* species. Various studies conducted at different hospitals around the globe have found a higher incidence of candidemia due to *C. parapsilosis* and several outbreaks have been caused by *C. parapsilosis* isolates which gradually increased from 2000 to 2021 [ 17 - 20 ]. *Candida parapsilosis* is one of the most prevalent species that causes candidemia worldwide, representing more than 20% of *Candida* species found in blood cultures in India, Brazil, Argentina, Peru, Spain, Russia, and China as well as more than 30% in Turkey, Greece, Croatia, Romania, South Africa, Nigeria, and Paraguay [ 21 ]. In India, *Candida* tropicalis is the most common species isolated from clinical specimens, including blood [ 22 ]. However, according to recent studies, there is an increase in the prevalence of infections caused by *C. parapsilosis* species and it is an emerging cause of candidemia in India. In a study performed by Tak et al., [ 23 ] in an Indian trauma care center, *C. parapsilosis* was the second most predominant pathogen causing 20% of all candidemia cases. A large study conducted on critically ill patients in Indian intensive care units showed *C. parapsilosis* species complex as the second most common cause of candidemia [ 24 ]. 

The prevalence of resistance in the present study was higher (16.7%), compared to previous studies by Singh et al. [ 25 ] (6.7%) in India. In a recent study, clinical isolates of *C. parapsilosis* sensu stricto were collected from nine hospitals during a 3-year surveillance study in India. It was the first comprehensive report documenting alarmingly high rates of fluconazole resistance in *C. parapsilosis* sensu stricto isolates in India. Overall, 32% of isolates were not susceptible (resistant and susceptible dose-dependent isolates) to fluconazole. In India, the rate of fluconazole resistance in *C. parapsilosis* varies, ranging from 12% to 76% in different hospitals [ 26 ]. 

In the present study, the results of antifungal susceptibility testing indicated that three (16.7%) *C. parapsilosis* isolates were resistant to fluconazole. Sequencing of the *ERG11* gene indicated that the underlying mechanism of resistance in these isolates was single nucleotide polymorphism. This caused an A395T mutation that led to an amino acid substitution Y132F in the *ERG11* gene. A recent study on *Candida* species from blood cultures collected from 16 hospitals located in the Madrid metropolitan area (2019-2021) reported that fluconazole resistance among *C. parapsilosis* reached as high as 13.6% and the resistant isolates harbored the dominant Y132F substitution [ 27 ]. Another study performed by Castanheira et al. [ 28 ] evaluated the fluconazole non-susceptible *C. parapsilosis* isolates collected from 2016 to 2017 in 60 hospitals located in 25 countries worldwide. In the aforementioned study, it was observed that most isolates carried the substitution Y132F in *ERG11* alone or with other resistance mechanisms. In the present study, there were three fluconazole-resistant isolates and all of them showed the Y132F mutation which was similar to a study in Brazil with nine isolates of fluconazole resistance and all showed the Y132F mutation [ 16 ]. In the present study, since the Y132F mutation was the most common cause of fluconazole resistance, a PCR assay was developed for rapid detection of this mutation among *C. parapsilosis* isolates. The total turnaround time for this PCR assay is 3 h. The developed PCR assay accurately detected Y132F mutation using in-house primers. Whereas all fluconazole-susceptible (n=15) *C. parapsilosis* isolates without Y132F mutation did not produce any band. The results of our assay 100% correlated with the results of AFST. Further sequencing of the *ERG11* gene revealed the presence of the target point mutation (Y132F) only in fluconazole-resistant *C. parapsilosis* isolates, thereby validating the PCR specificity. This shows that our PCR assay findings were 100% consistent with DNA sequencing results.

The limitation of this study was that the developed PCR assay was specific to one of the resistant mechanisms of fluconazole in *C. parapsilosis* in which a single point mutation (A395T) of the *ERG11* gene led to an amino acid substitution (Y132F). This mechanism was targeted since it was the most prevalent mechanism of resistance. Since this study was conducted in a single center, the number of fluconazole-resistant *C. parapsilosis* isolates included in this study was small. A multicentric study with a large number of resistant isolates involving other resistant mechanisms would help in the authentication of the findings.

## Conclusion

This in-house PCR can detect fluconazole resistance with 100% sensitivity and specificity in less than 3 h by mainly targeting the Y132F mutation which was the most common mutation. Hence, this assay can be used as an early method for the detection of fluconazole resistance among *C. parapsilosis* isolates which has become highly prevalent and this will also help the provision of appropriate antifungal treatment for better patient outcomes.
